# Supplementary material for: A Humanized Yeast Phenomic Model of Deoxycytidine Kinase to Predict Genetic Buffering of Nucleoside Analog Cytotoxicity
Source: Genes (Basel). 2019 Sep 30;10(10):770. doi: 10.3390/genes10100770 (PMC6826991; doi:10.3390/genes10100770)
Supplement: Supplementary file 1 [file genes-10-00770-s001.zip › Additional_Files/Additional_File7_GOTermHeatmaps.zip!/Additional_File7_GOTermHeatmaps/cleavage_involved_in_rRNA_processing.pdf]

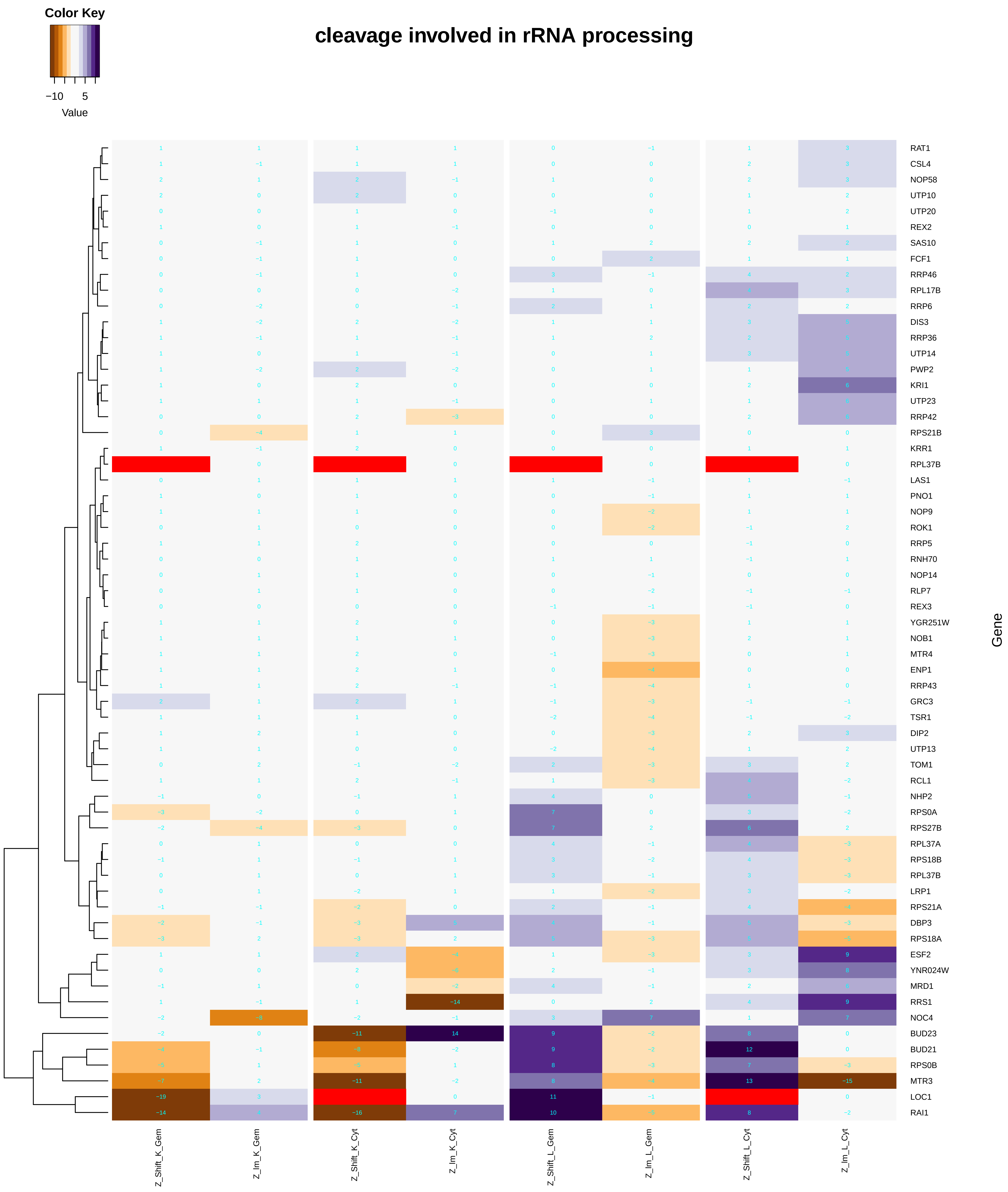
cleavage involved in rRNA processing
Color Key
−10
5
Value
Gene
RAT1
CSL4
NOP58
UTP10
UTP20
REX2
SAS10
FCF1
RRP46
RPL17B
RRP6
DIS3
RRP36
UTP14
PWP2
KRI1
UTP23
RRP42
RPS21B
KRR1
RPL37B
LAS1
PNO1
NOP9
ROK1
RRP5
RNH70
NOP14
RLP7
REX3
YGR251W
NOB1
MTR4
ENP1
RRP43
GRC3
TSR1
DIP2
UTP13
TOM1
RCL1
NHP2
RPS0A
RPS27B
RPL37A
RPS18B
RPL37B
LRP1
RPS21A
DBP3
RPS18A
ESF2
YNR024W
MRD1
RRS1
NOC4
BUD23
BUD21
RPS0B
MTR3
LOC1
RAI1
Z_Shift_K_Gem
Z_Im_K_Gem
Z_Shift_K_Cyt
Z_Im_K_Cyt
Z_Shift_L_Gem
Z_Im_L_Gem
Z_Shift_L_Cyt
Z_Im_L_Cyt

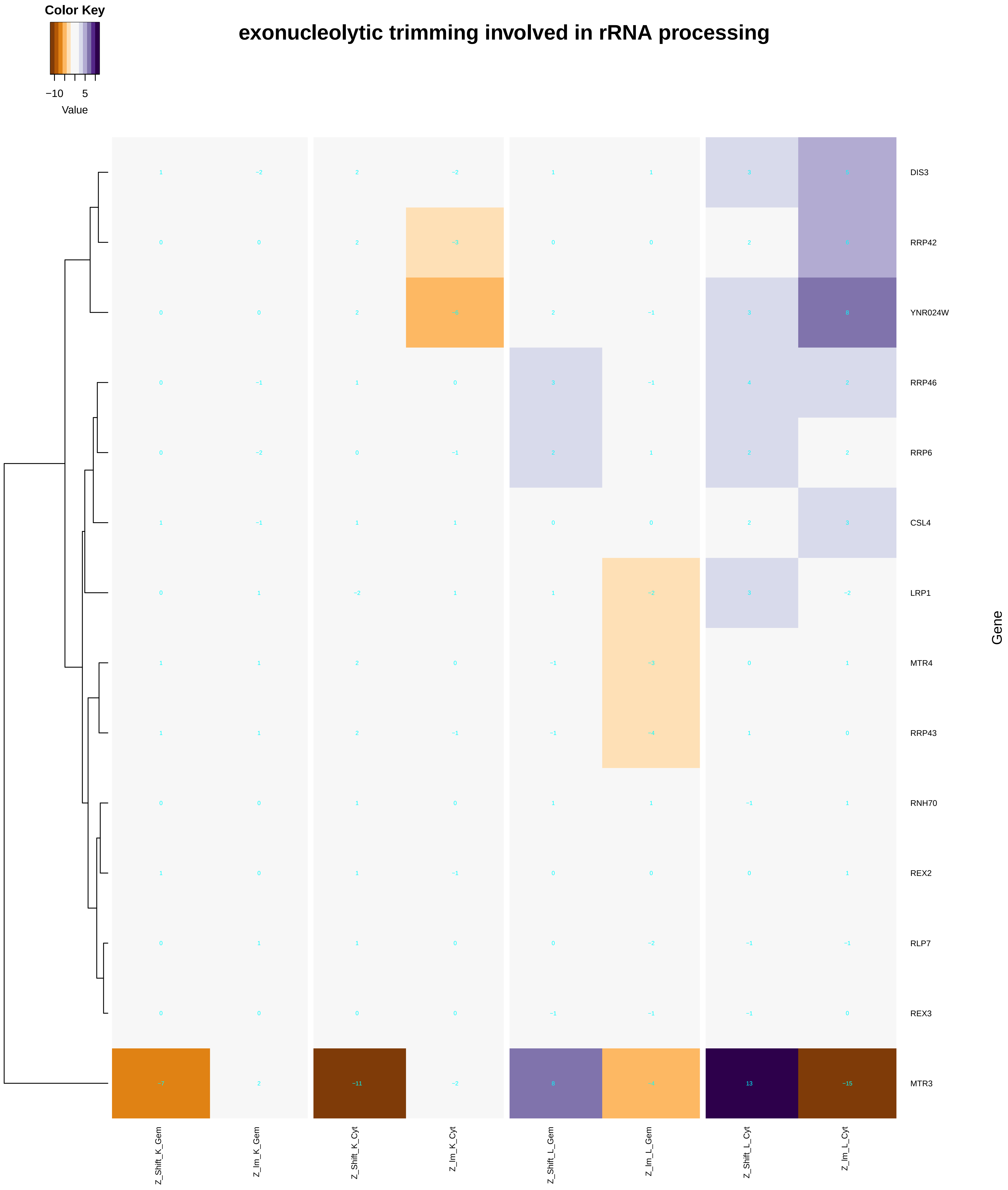
exonucleolytic trimming involved in rRNA processing
Color Key
−10
5
Value
DIS3
RRP42
YNR024W
RRP46
RRP6
CSL4
LRP1
MTR4
RRP43
RNH70
REX2
RLP7
REX3
MTR3
Gene
Z_Shift_K_Gem
Z_Im_K_Gem
Z_Shift_K_Cyt
Z_Im_K_Cyt
Z_Shift_L_Gem
Z_Im_L_Gem
Z_Shift_L_Cyt
Z_Im_L_Cyt

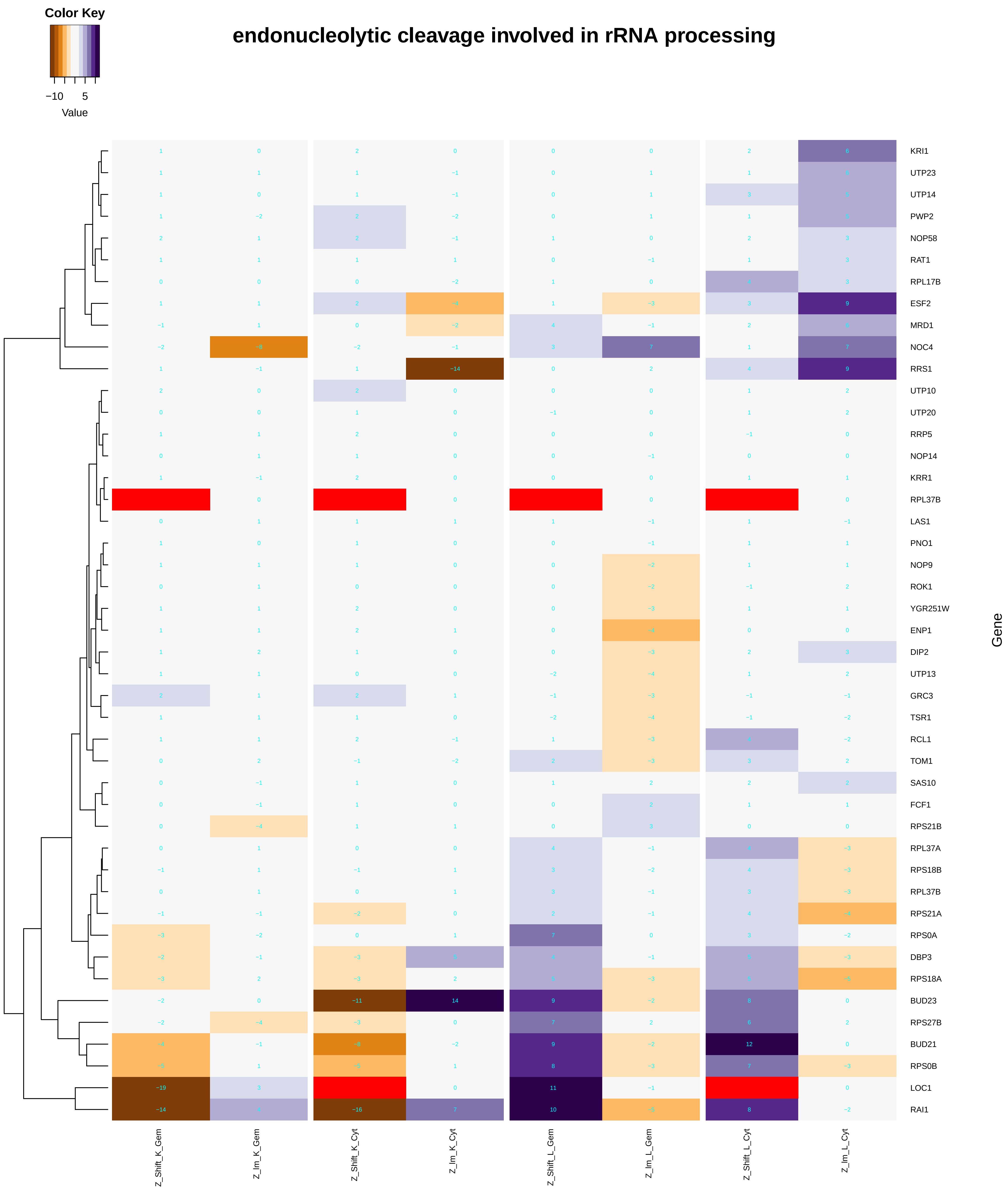

endonucleolytic cleavage involved in rRNA processing
Color Key
−10
5
Value
KRI1
UTP23
UTP14
PWP2
NOP58
RAT1
RPL17B
ESF2
MRD1
NOC4
RRS1
UTP10
UTP20
RRP5
NOP14
KRR1
RPL37B
LAS1
PNO1
NOP9
ROK1
YGR251W
ENP1
DIP2
UTP13
GRC3
TSR1
RCL1
TOM1
SAS10
FCF1
RPS21B
RPL37A
RPS18B
RPL37B
RPS21A
RPS0A
DBP3
RPS18A
BUD23
RPS27B
BUD21
RPS0B
LOC1
RAI1
Gene
Z_Shift_K_Gem
Z_Im_K_Gem
Z_Shift_K_Cyt
Z_Im_K_Cyt
Z_Shift_L_Gem
Z_Im_L_Gem
Z_Shift_L_Cyt
Z_Im_L_Cyt

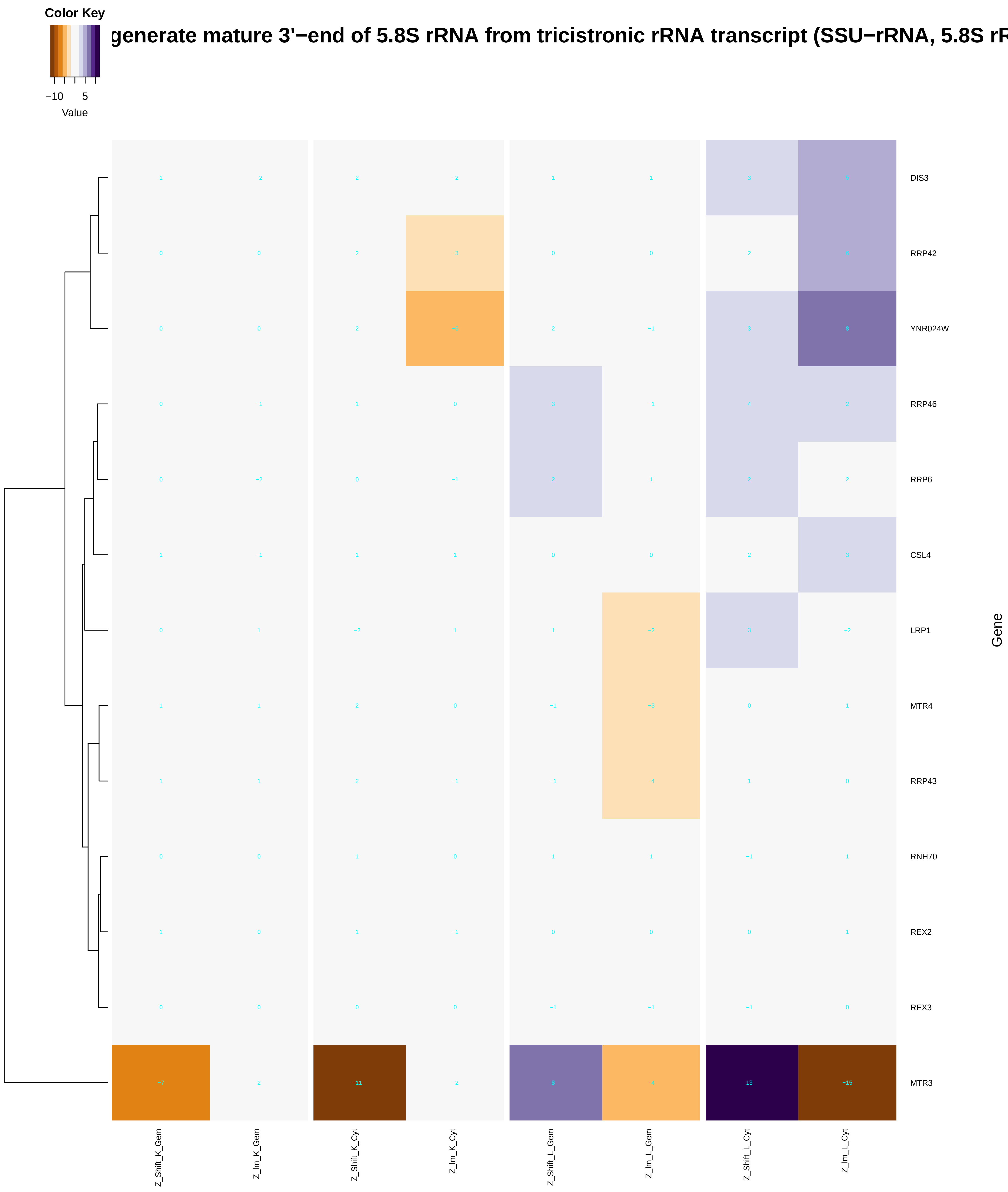

generate mature 3'−end of 5.8S rRNA from tricistronic rRNA transcript (SSU−rRNA, 5.8S rR
Color Key
−10
5
Value
DIS3
RRP42
YNR024W
RRP46
RRP6
CSL4
LRP1
MTR4
RRP43
RNH70
REX2
REX3
MTR3
Gene
Z_Shift_K_Gem
Z_Im_K_Gem
Z_Shift_K_Cyt
Z_Im_K_Cyt
Z_Shift_L_Gem
Z_Im_L_Gem
Z_Shift_L_Cyt
Z_Im_L_Cyt

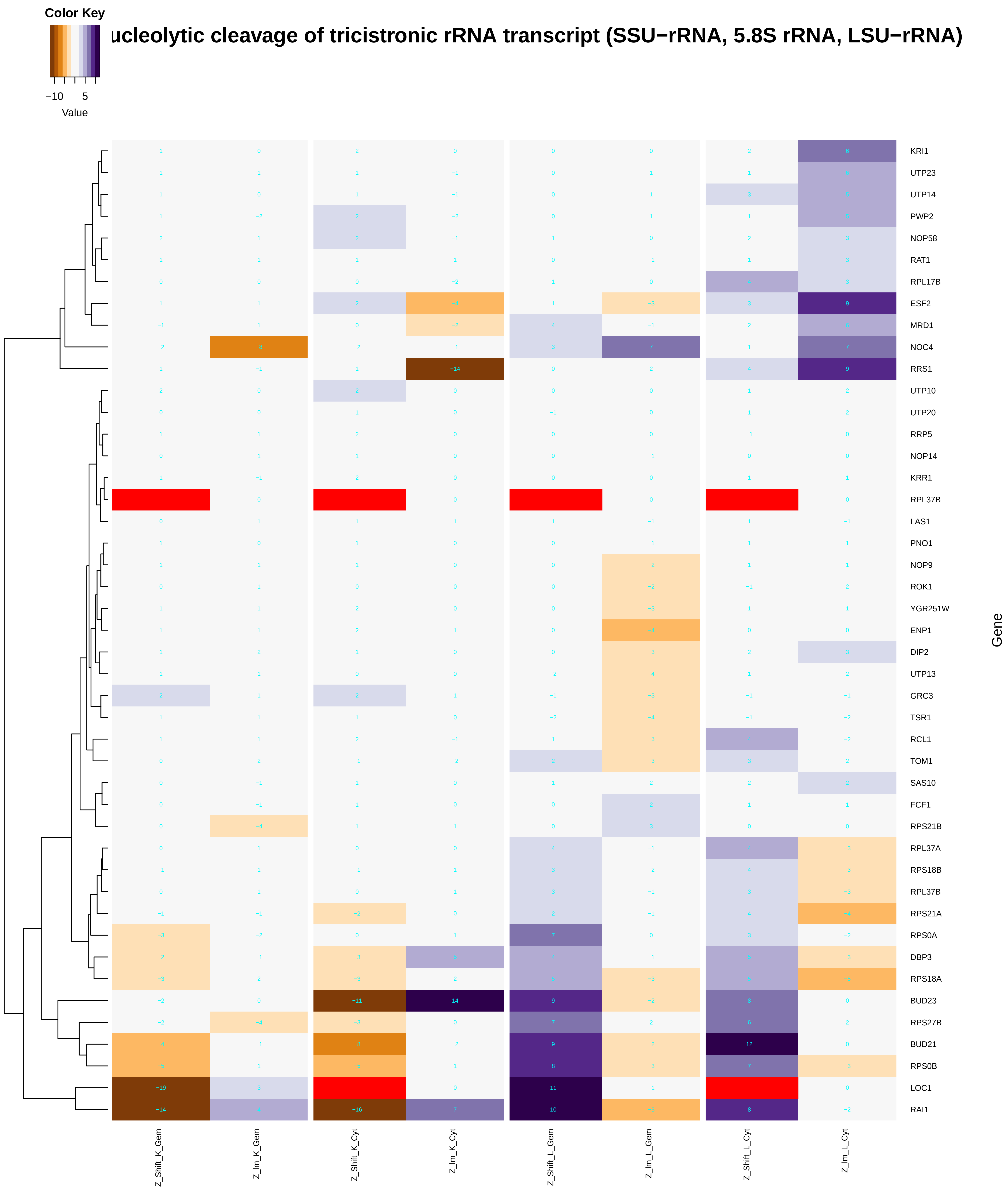

Color Key
−10
5
Value
ucleolytic cleavage of tricistronic rRNA transcript (SSU−rRNA, 5.8S rRNA, LSU−rRNA)
KRI1
UTP23
UTP14
PWP2
NOP58
RAT1
RPL17B
ESF2
MRD1
NOC4
RRS1
UTP10
UTP20
RRP5
NOP14
KRR1
RPL37B
LAS1
PNO1
NOP9
ROK1
YGR251W
ENP1
DIP2
UTP13
GRC3
TSR1
RCL1
TOM1
SAS10
FCF1
RPS21B
RPL37A
RPS18B
RPL37B
RPS21A
RPS0A
DBP3
RPS18A
BUD23
RPS27B
BUD21
RPS0B
LOC1
RAI1
Gene
Z_Shift_K_Gem
Z_Im_K_Gem
Z_Shift_K_Cyt
Z_Im_K_Cyt
Z_Shift_L_Gem
Z_Im_L_Gem
Z_Shift_L_Cyt
Z_Im_L_Cyt

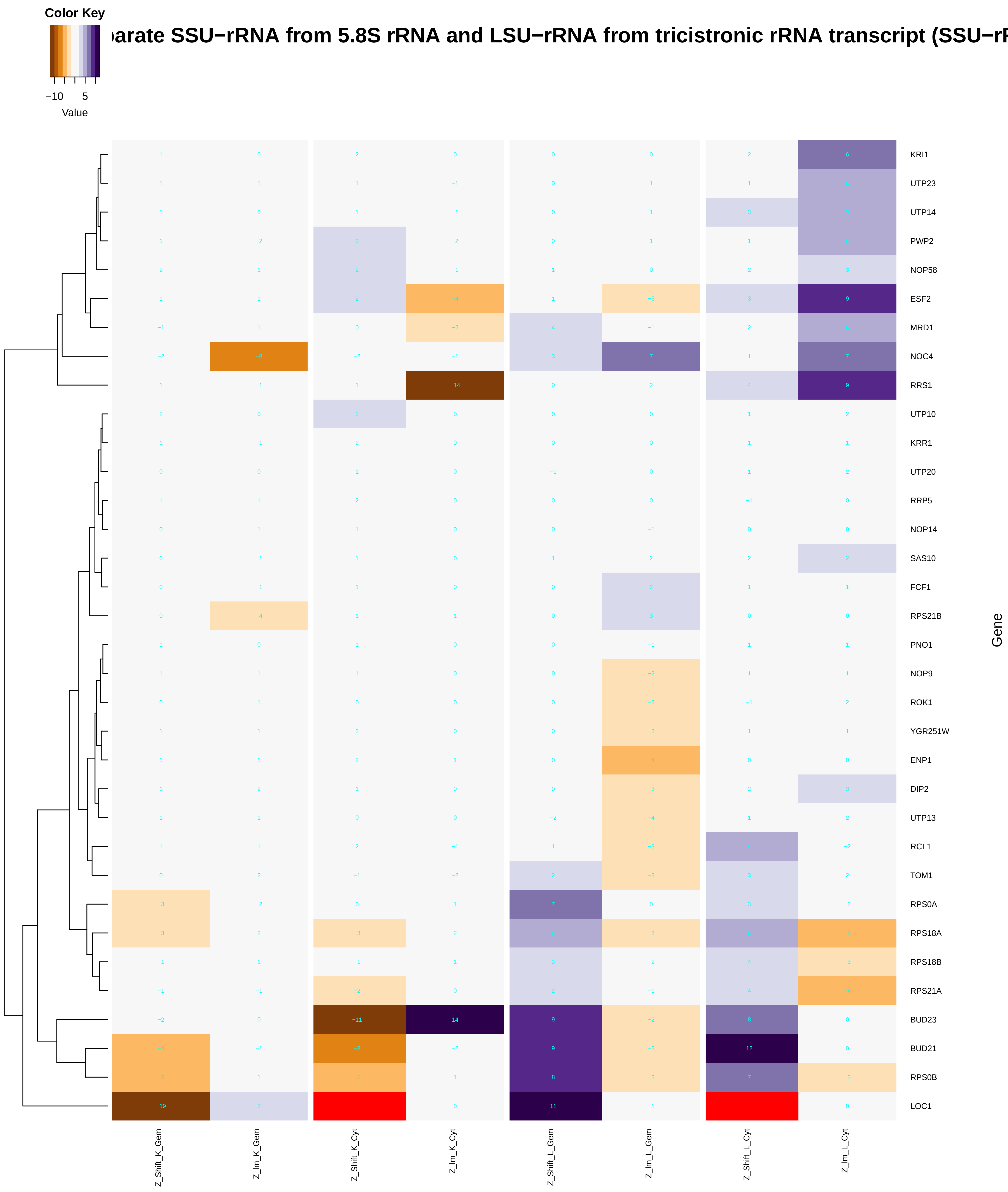
Color Key
−10
5
Value
›arate SSU−rRNA from 5.8S rRNA and LSU−rRNA from tricistronic rRNA transcript (SSU−rR
KRI1
UTP23
UTP14
PWP2
NOP58
ESF2
MRD1
NOC4
RRS1
UTP10
KRR1
UTP20
RRP5
NOP14
SAS10
FCF1
RPS21B
PNO1
NOP9
ROK1
YGR251W
ENP1
DIP2
UTP13
RCL1
TOM1
RPS0A
RPS18A
RPS18B
RPS21A
BUD23
BUD21
RPS0B
LOC1
Gene
Z_Shift_K_Gem
Z_Im_K_Gem
Z_Shift_K_Cyt
Z_Im_K_Cyt
Z_Shift_L_Gem
Z_Im_L_Gem
Z_Shift_L_Cyt
Z_Im_L_Cyt

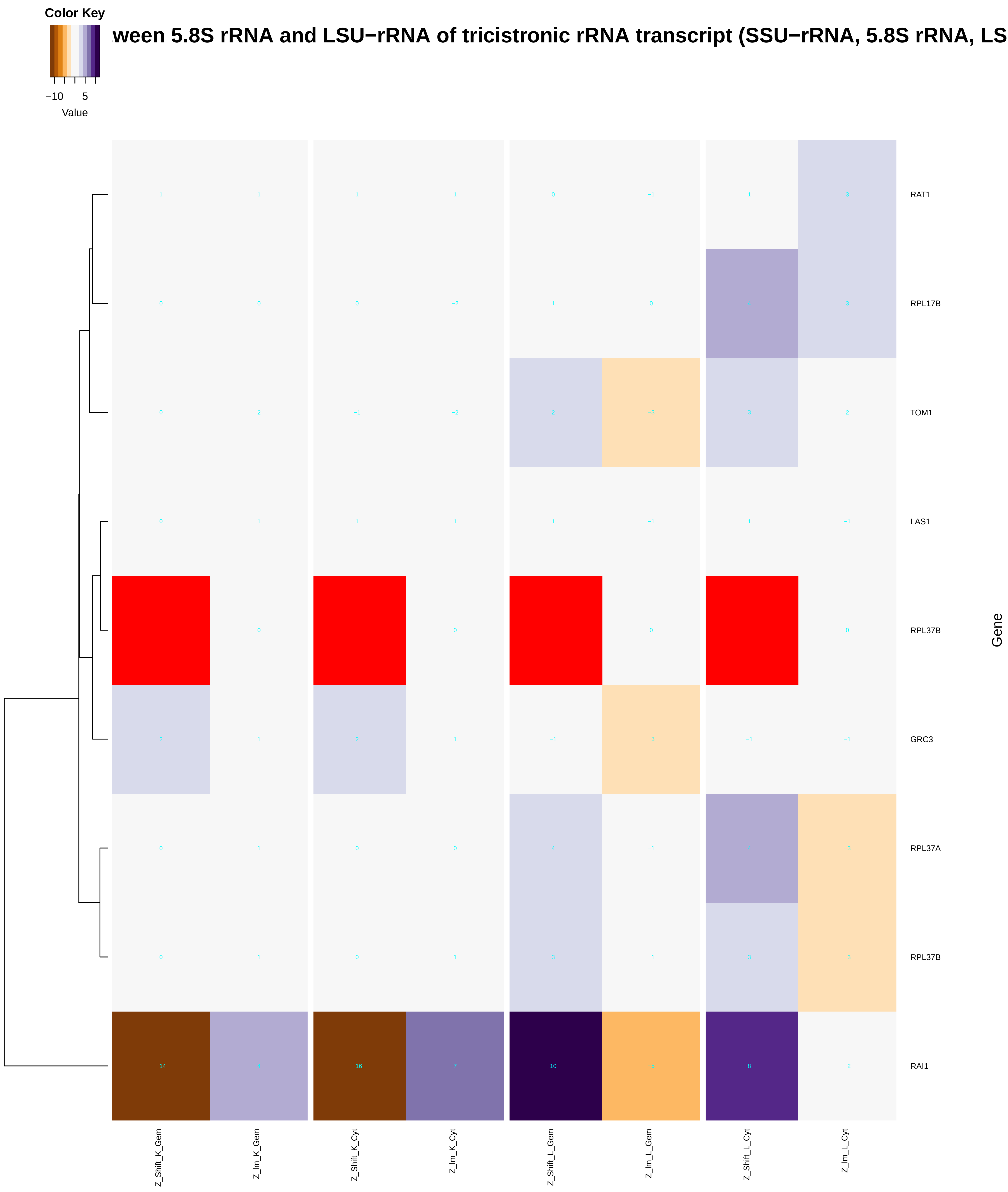

ween 5.8S rRNA and LSU−rRNA of tricistronic rRNA transcript (SSU−rRNA, 5.8S rRNA, LS
Color Key
−10
5
Value
RAT1
RPL17B
TOM1
LAS1
RPL37B
GRC3
RPL37A
RPL37B
RAI1
Gene
Z_Shift_K_Gem
Z_Im_K_Gem
Z_Shift_K_Cyt
Z_Im_K_Cyt
Z_Shift_L_Gem
Z_Im_L_Gem
Z_Shift_L_Cyt
Z_Im_L_Cyt

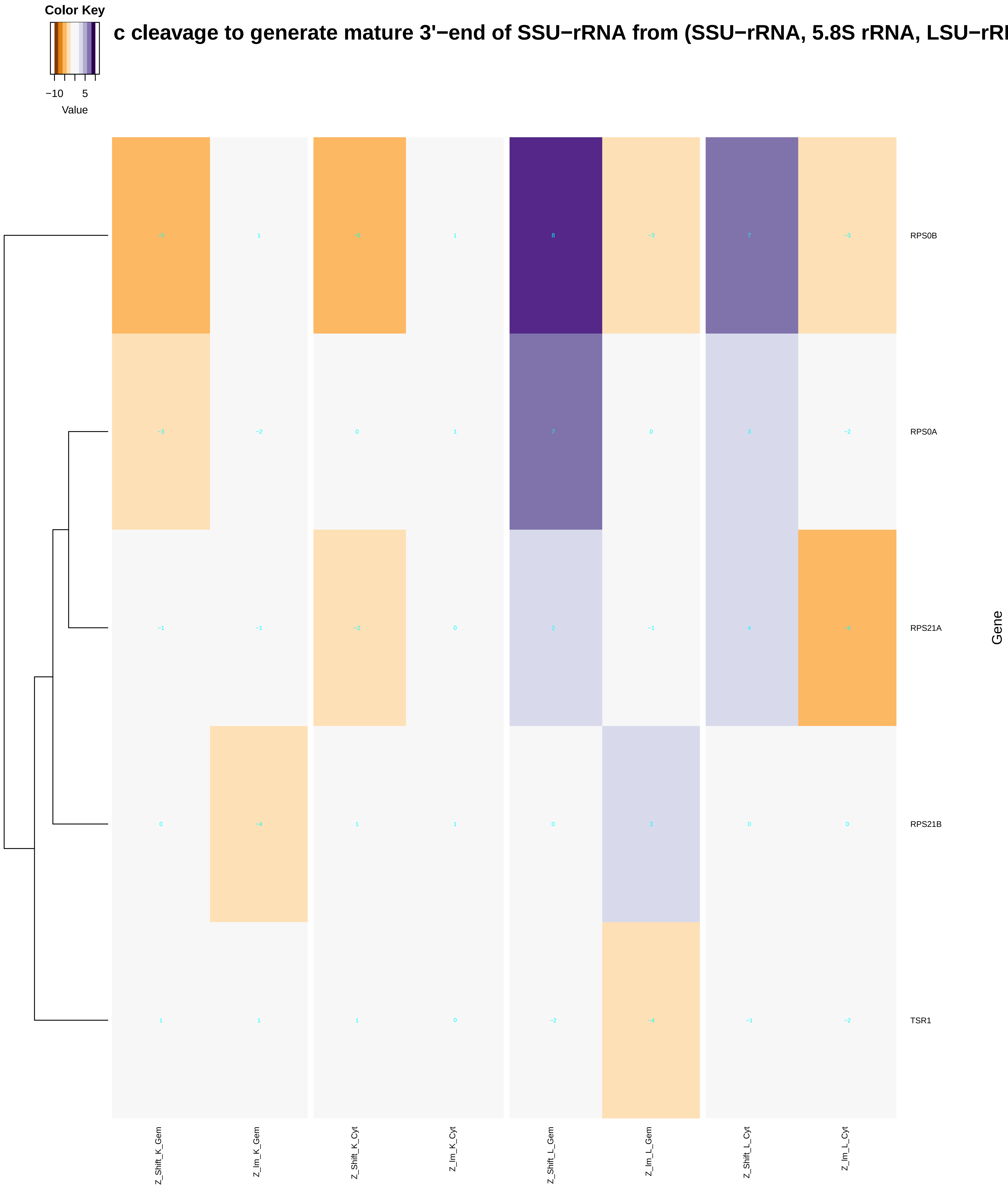

Color Key
−10
5
Value
c cleavage to generate mature 3'−end of SSU−rRNA from (SSU−rRNA, 5.8S rRNA, LSU−rR
RPS0B
RPS0A
RPS21A
RPS21B
TSR1
Gene
Z_Shift_K_Gem
Z_Im_K_Gem
Z_Shift_K_Cyt
Z_Im_K_Cyt
Z_Shift_L_Gem
Z_Im_L_Gem
Z_Shift_L_Cyt
Z_Im_L_Cyt

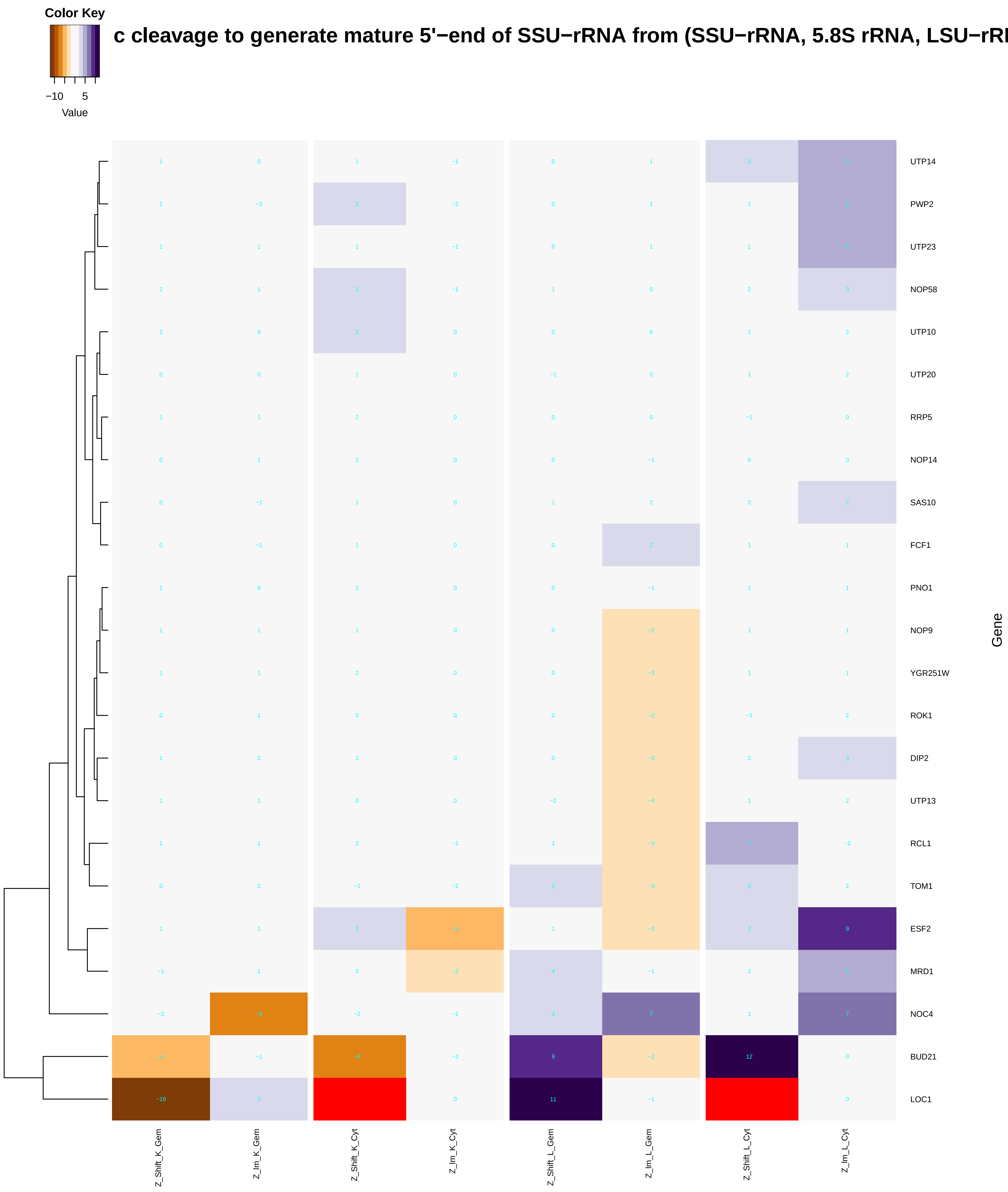

Color Key
c cleavage to generate mature 5'−end of SSU−rRNA from (SSU−rRNA, 5.8S rRNA, LSU−rRI
−10
5
Value
UTP14
PWP2
UTP23
NOP58
UTP10
UTP20
RRP5
NOP14
SAS10
FCF1
PNO1
NOP9
YGR251W
ROK1
DIP2
UTP13
RCL1
TOM1
ESF2
MRD1
NOC4
BUD21
LOC1
Gene
Z_Shift_K_Gem
Z_Im_K_Gem
Z_Shift_K_Cyt
Z_Im_K_Cyt
Z_Shift_L_Gem
Z_Im_L_Gem
Z_Shift_L_Cyt
Z_Im_L_Cyt

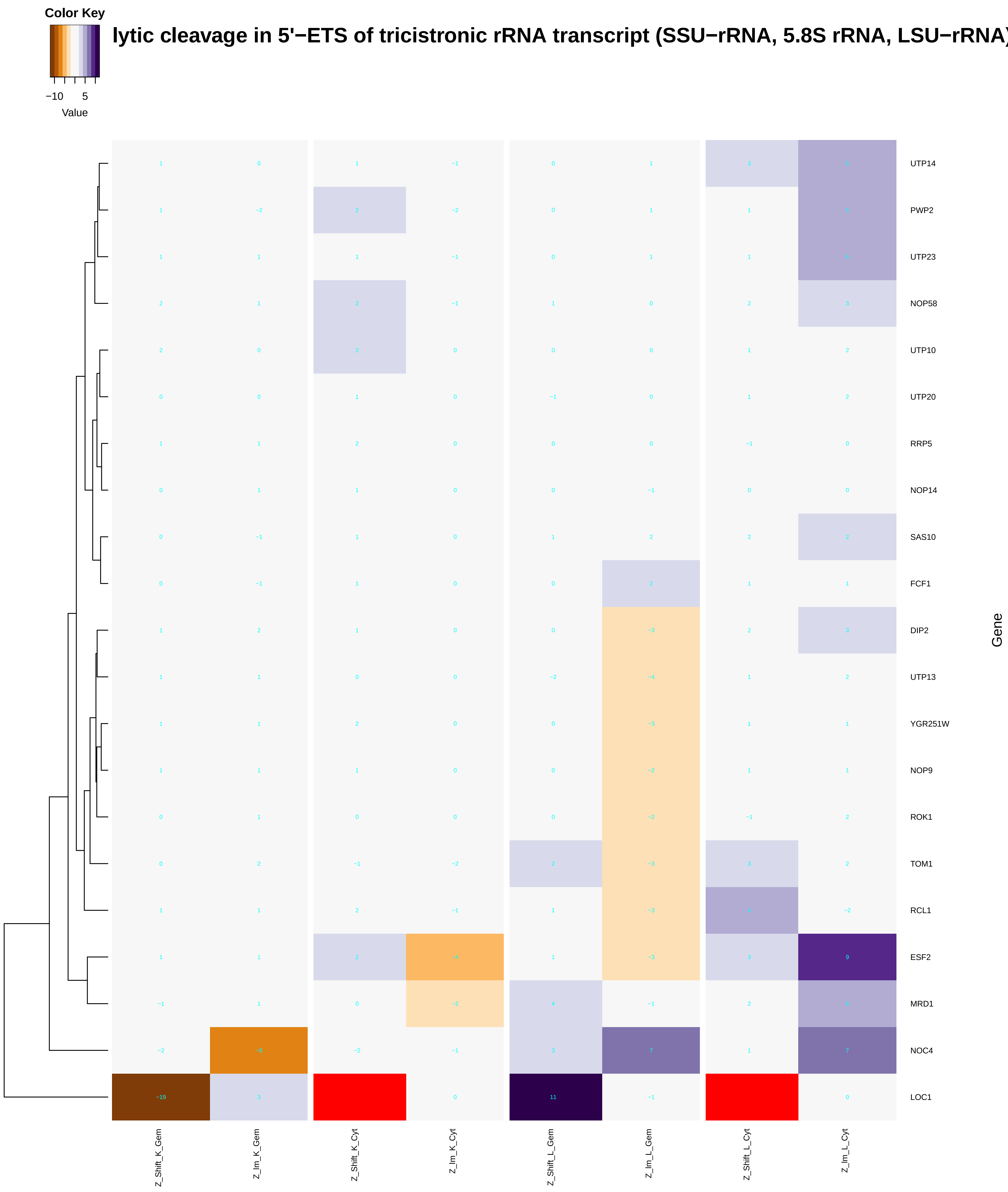

Color Key
−10
5
Value
lytic cleavage in 5'−ETS of tricistronic rRNA transcript (SSU−rRNA, 5.8S rRNA, LSU−rRNA
UTP14
PWP2
UTP23
NOP58
UTP10
UTP20
RRP5
NOP14
SAS10
FCF1
DIP2
UTP13
YGR251W
NOP9
ROK1
TOM1
RCL1
ESF2
MRD1
NOC4
LOC1
Gene
Z_Shift_K_Gem
Z_lm_K_Gem
Z_Shift_K_Cyt
Z_lm_K_Cyt
Z_Shift_L_Gem
Z_lm_L_Gem
Z_Shift_L_Cyt
Z_lm_L_Cyt
